# Anodal tDCS over the Primary Motor Cortex Facilitates Long-Term Memory Formation Reflecting Use-Dependent Plasticity

**DOI:** 10.1371/journal.pone.0127270

**Published:** 2015-05-21

**Authors:** Orjon Rroji, Kris van Kuyck, Bart Nuttin, Nicole Wenderoth

**Affiliations:** 1 Department of Kinesiology, Research Center for Movement Control and Neuroplasticity, KU Leuven, Leuven, Belgium; 2 Department of Neurosciences, Research Group Experimental Neurosurgery and Neuroanatomy, KU Leuven, Leuven, Belgium; 3 Department of Health Sciences and Technology, Neural Control of Movement Laboratory, ETH Zurich, Zürich, Switzerland; University of Waterloo, CANADA

## Abstract

Previous research suggests that anodal transcranial direct current stimulation (tDCS) over the primary motor cortex (M1) modulates NMDA receptor dependent processes that mediate synaptic plasticity. Here we test this proposal by applying anodal versus sham tDCS while subjects practiced to flex the thumb as fast as possible (ballistic movements). Repetitive practice of this task has been shown to result in performance improvements that reflect use-dependent plasticity resulting from NMDA receptor mediated, long-term potentiation (LTP)-like processes. Using a double-blind within-subject cross-over design, subjects (n=14) participated either in an anodal or a sham tDCS session which were at least 3 months apart. Sham or anodal tDCS (1 mA) was applied for 20 min during motor practice and retention was tested 30 min, 24 hours and one week later. All subjects improved performance during each of the two sessions (p < 0.001) and learning gains were similar. Our main result is that long term retention performance (i.e. 1 week after practice) was significantly better when practice was performed with anodal tDCS than with sham tDCS (p < 0.001). This effect was large (Cohen’s d=1.01) and all but one subject followed the group trend. Our data strongly suggest that anodal tDCS facilitates long-term memory formation reflecting use-dependent plasticity. Our results support the notion that anodal tDCS facilitates synaptic plasticity mediated by an LTP-like mechanism, which is in accordance with previous research.

## Introduction

Transcranial Direct Current Stimulation (tDCS) is a non-invasive and well-tolerated brain stimulation technique that can be applied to cortical areas [1]. tDCS modulates spontaneous neuronal network activity [2] by injecting a low amplitude direct current that passes between surface electrodes placed on the scalp. Anodal tDCS applied to the human primary motor cortex (M1) induces measurable changes in corticomotor excitability that last beyond the stimulation period, which is commonly referred to as an after-effect [3]. Numerous studies have shown that anodal tDCS over M1 combined with motor practice facilitates motor learning in healthy volunteers [4–16] even when applied during a single training session [17–19]. However, it is still not fully understood how tDCS after-effects might facilitate motor learning.

It has been hypothesized that tDCS after-effects are synaptically driven, depend on the glutamatergic system and might be mediated by a long-term potentiation (LTP)-like mechanism. These suppositions are supported by work in both human and animal models. tDCS after-effects in human are abolished when NMDA receptors are blocked [11], while facilitating NMDA receptor activity prolongs the increase in excitability caused by anodal tDCS [20]. When applied to mouse M1 slices, anodal tDCS induces long-lasting synaptic potentiation that is NMDA receptor dependent [21]. Additionally, free GABA is reduced after anodal tDCS [12] and GABAergic inhibition is released [22–24]. The reduction of GABAergic inhibition is believed to have a “gating function” to increase (glutamatergic) plasticity [25]. These effects increase the probability of LTP occurring at those synapses that are activated by behavioral processes such as motor training. Furthermore, it has also been suggested that the plasticity enhancing effect of anodal tDCS is mediated by brain derived neurotrophic factor (BDNF) dependent mechanisms which are important for structural changes at the synaptic level that promote long term consolidation [21].

While the cellular mechanisms of synaptic plasticity can be directly tested in animal models, in human they can only be indirectly inferred. One paradigm that has been shown to activate LTP-like mechanisms in human is the repeated practice of motor actions that induces neural changes known as use-dependent plasticity. For example, after several minutes of brisk thumb movements use-dependent plasticity is clearly evident [26,27]. Such training is believed to strengthen existing neural connections and to facilitate the creation of new ones within M1 [28]. Moreover, pharmacological studies have shown that its expression depends on NMDA receptor activity [29] and that effects are enhanced when GABAergic inhibition is reduced.

In summary, previous research strongly suggests that anodal tDCS over M1 acts on cellular pathways that mediate use-dependent plasticity and should therefore facilitate learning. We tested this hypothesis by applying anodal tDCS during a single training session of a ballistic thumb movement task which was followed by several retention tests that were executed 30 min, 24 hours and one week after practice had finished.

In accordance to previous work using this or similar motor tasks [27,30–33] we quantified use-dependent plasticity by changes of movement kinematics (here thumb velocity). Our underlying theoretical model is that the brain optimizes its forward command resulting in a more efficient muscle activation pattern, thus agonistic muscles are activated in a more synchronized manner (e.g. by augmenting the descending drive) while antagonists are more effectively inhibited. This will result in higher velocities/acceleration of the movement particularly for simple tasks. This theoretical model is compatible with current views suggesting that M1 neurons represent primarily kinematics rather than kinetics [34] and that training improves central representations of these movement patterns [35].

We used a double-blind within-subject cross-over design where subjects practiced ballistic thumb movements while either anodal tDCS or sham tDCS was applied during two separate sessions that were at least 3 months apart. The cross-over design was chosen to reduce the influence of inter-individual differences in ability to undergo practice related neuroplastic changes, which can vary substantially and might result from the genetic background of the individual [36] or previous motor experience [30].

## Materials and Methods

### Participants

Eighteen young healthy volunteers were recruited for this study. Four subjects did drop out for personal reasons before performing the cross-over test and were excluded from all analyses. The remaining 14 subjects were between 18–29 years of age (mean age = 23 ± 7 years, 7 male). Ten subjects were right-handed (Edinburgh Handedness Inventory) [37]. None of the subjects had prior experience with the motor task and all were naïve to the purpose of the experiment. Subjects provided written informed consent prior to participation and were reimbursed. All experimental procedures were approved by the Ethics Committee for Biomedical Research at the Katholieke Universiteit Leuven (ethics approval number: S52763) in accordance with the Code of Ethics of the World Medical Association (Declaration of Helsinki) [38].

### Motor task

**Fig 1:**
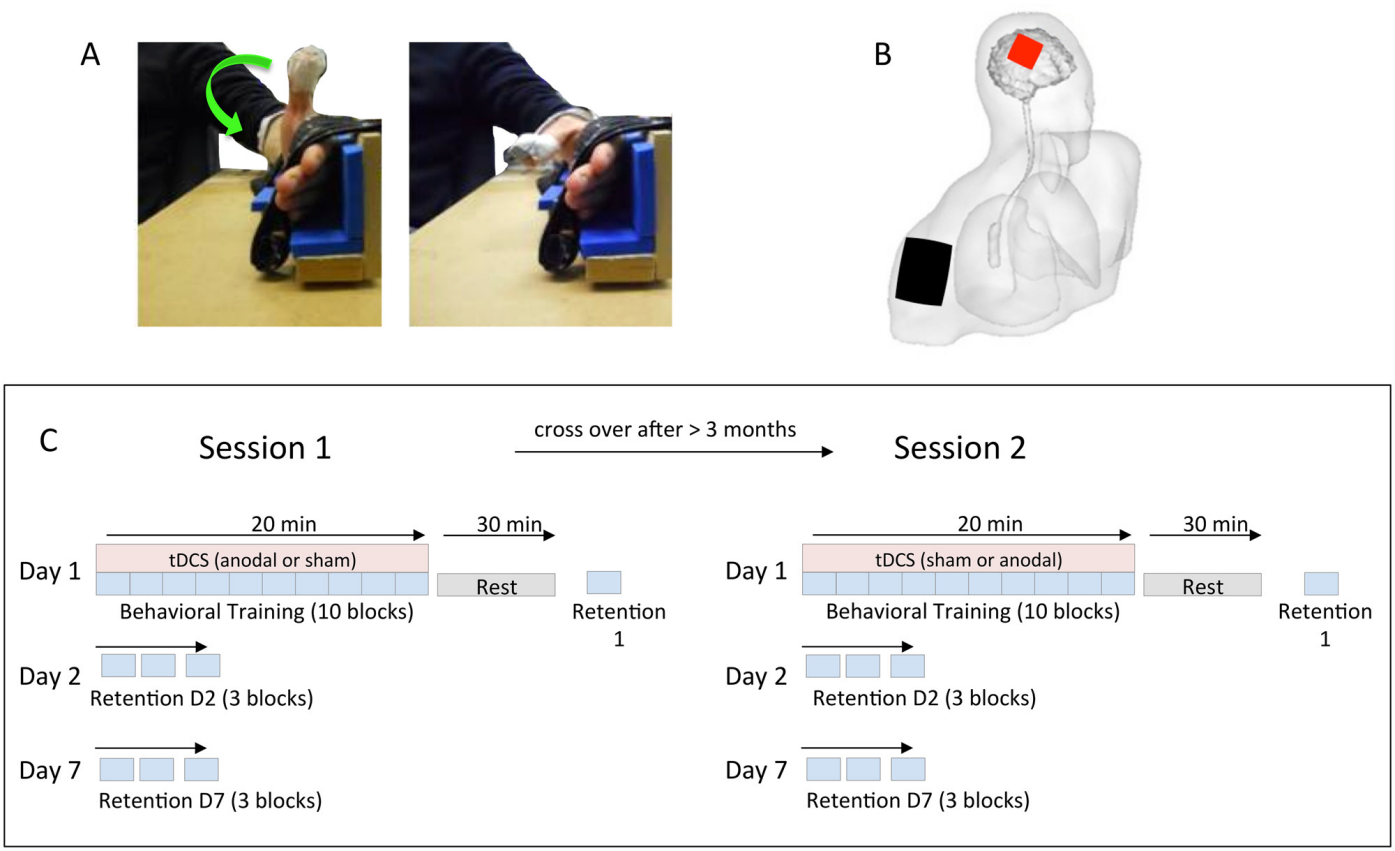
Experimental Setup. A) Subjects performed discrete ballistic thumb flexion movements with the forearm and fingers fixated. B) Constant current stimulation was delivered with the anode (red) placed over the M1 contralateral to the moving thumb and the cathode (black) over the ipsilateral shoulder. C) General experimental design.

Subjects were seated in a comfortable chair and had to perform discrete ballistic thumb flexion movements with their non-dominant hand (Fig 1A). The forearm was fixed to a wooden construction and the four fingers were immobilized by a velcro strap while the thumb was unconstrained and could move freely. A Polhemus Fastrak sensor (sampling rate of 120 Hz, spatial resolution of 0.0006 cm) was fixed on the nail of the thumb to measure 3D kinematics and provide online feedback. This sensor location was used because previous research has shown that it is highly reproducible between sessions [31]. 3D kinematic data was used to calculate the absolute velocity: V_i_ = sqrt ((X_i_-X_i-1_)^2^ + (Y_i_-Y_i-1_)^2^ + (Z_i_-Z_i-1_)^2^) / (t_i_-t_i-1_) where *X*, *Y* and *Z* represent displacement in three dimensions, *t* the time and *i* the index of the current data point. For each movement the velocity profile was displayed on a computer screen in front of the subject to provide performance feedback. The maximum velocity was also displayed for each trial and continuously updated providing subjects with an indication of how their performance changed across training.

### tDCS

Transcranial direct current stimulation (tDCS) was delivered by a battery driven constant current stimulator (HDC stim part of HDC kit, Medical device CE 0068, Newronika s.r.l. Milan—Italy) which was connected to two rubber electrodes enclosed in saline soaked sponges (Fig 1B). The anode (5 x 5 cm) was located over the hand area of the M1, which was localized with transcranial magnetic stimulation (TMS). The general TMS procedure was nearly identical to that described in Alaerts et al., [39]. In short, electromyograms (EMG, Mespec 8000, Mega Electronics Ltd., Kuopio, Finland) were recorded with disposable Ag-AgCl surface electrodes (Blue Sensor SP, Denmark) from the abductor pollicis brevis (APB). Focal TMS was performed with a 70mm figure of eight magnetic coil connected to a Magstim 200 stimulator (Magstim, Whitland, Dyfed UK). The coil was positioned tangential to the scalp of the non-dominant hemisphere with the handle pointing backward at an angle of 45° away from the mid sagittal line. TMS was used to determine the so-called motor “hotspot”, i.e. the position where the largest and most consistent MEPs were obtained in the APB. The APB hotspot was marked on the scalp and the centre of the anodal electrode was positioned over this point. The average hotspot position was 5.2 ± 0.8 cm lateral to the midline and 0.9 ± 1.1 cm anterior to the intraural line. The cathode (11 x 9 cm) was located on the ipsilateral shoulder (extracephalic placement). We did not test TMS in 4 subjects because of technical problems (malfunctioning and repair of stimulator) and we placed the electrode 5 cm laterally from the cortex and 1 cm anterior to the intraaural line.

In the anodal tDCS condition the current was ramped up to 1.0 mA over 12 s and then applied at this intensity continuously for 20 min. In the sham tDCS condition the same ramp up procedure was applied, but the current was ramped down after 12 s (sham tDCS).

### Overall design

We employed a cross-over design with all subjects participating in anodal tDCS and sham tDCS sessions (order counterbalanced across subjects) which were at least 12 weeks apart (Fig 1C). Both subjects and the experimenter were blinded as to which stimulation was applied.

At the beginning of each session there was a short demonstration by the experimenter that was followed by 5 warm-up trials. Ten practice blocks were then executed (train 1…train 10) each consisting of 20 flexion movements (1 trial every 3 s). One practice block lasted 1 min in total and was followed by a 1 min break to prevent fatigue. The total training session lasted 20 min (corresponding to 200 flexion movements) and during this time either anodal tDCS or sham tDCS was administered. After training the tDCS electrodes were removed and subjects rested for 30 min. A retention test (RT-D1) was then performed consisting of 1 block of 20 flexion movements. Additional retention tests were performed the following day (RT-D2) and one week (RT-D7) later, each consisting of 3 blocks of 20 flexion movements (S1 File).

At the end of the experiment subjects were debriefed. None reported suffering serious headaches, nausea or pain. Even though some subjects reported an initial tingling sensation they perceived no difference between the two sessions, which was likely due to the fact that sessions were at least 3 months apart.

### Statistics

Since a cross-over design was used, i.e. subjects had to perform the motor training twice, we first examined the influence of session-effects on performance improvement. Peak velocities were averaged within each block. All blocks were normally distributed (Shapiro-Wilk test, p > 0.055) and the assumption of sphericity was met (Mauchly’s test of sphericity). We tested for potential session-effects by entering these data into an analysis of variance for repeated measurements (repeated measures ANOVA) with the within-subjects factors *session* (1, 2) and *block* (train1…train10).

Next we tested the effect of anodal versus sham tDCS on use-dependent plasticity. For each session performance was normalized to the first training block (performance improvement_1…10_ (%) = ((peak velocity_1…10_ / train1) * 100) (S1 File). Data were normally distributed, except for one block (p = 0.042 for train 5 of the anodal tDCS session), and the assumption of sphericity was met. The % performance improvement data were entered into a repeated measures ANOVA with the within-subjects factors *stimulation* (anodal tDCS, sham tDCS) and *block* (train2…train10, RT-D1-1, RT-D2-1… RT-D2-3, RT-D7-1… RT-D7-3) (, and the between-subjects factor *order* (anodal-sham, sham-anodal). The alpha level was α = 0.05 and Fischer’s LSD *post-hoc* tests were used to analyze significant interaction effects.

Finally, for significant *stimulation* x *block* interaction effects Cohen’s d (effect size for dependent measurements) was calculated. Further details are described in the results section. All results in the text are reported as mean (M) and standard deviation (STD). Error bars in the figures display the standard error of the mean (SEM).

## Results

**Fig 2:**
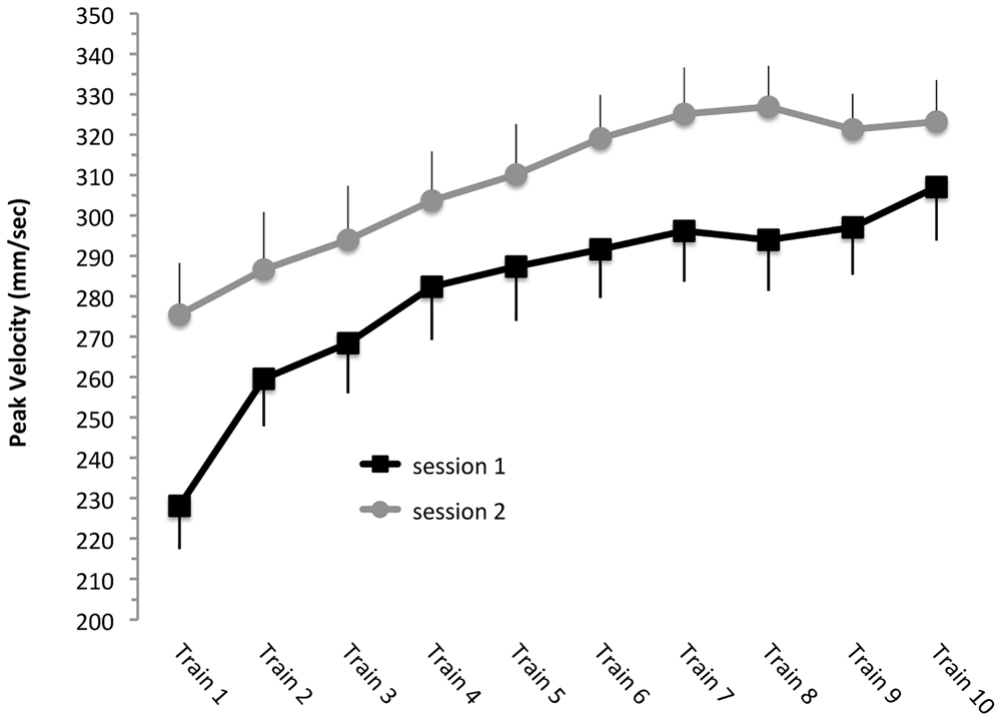
Order effects. Peak velocity data of the practice blocks (train1…10) performed in session 1 (black squares) and session 2 (gray circles). Note that when data are collapsed across anodal tDCS and sham tDCS conditions peak velocity was generally higher in the second session, but the extent of improvement over the course of learning was similar. Data are shown as M ± SEM.

Training resulted in a reliable increase in thumb flexion peak velocity which was observed for each session (Fig 2; main effect of *block* F(16, 208) = 19.20, p < 0.0001; note that for each session data was collapsed across anodal tDCS and sham tDCS conditions). Not surprisingly, overall peak velocities were significantly higher in the second than the first session (main effect of *session*: F(1, 12) = 11.30, p < 0.005). Importantly, the learning gains (indicating that use-dependent plasticity took place) were similar across sessions (*session* x *block* interaction: F(16, 208) = 0.81, p = 0.675), i.e. we found no statistical evidence indicating that subjects learned more in the first than the second session (or vice versa).

**Fig 3:**
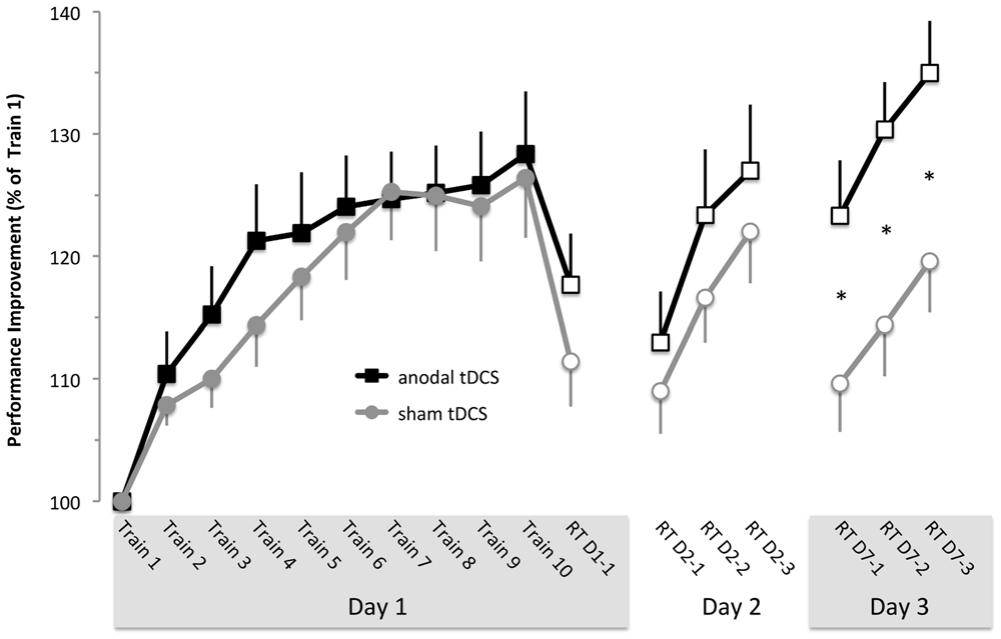
Stimulation effects. Performance improvements relative to the first training block for the anodal tDCS session (black squares) and the sham tDCS session (gray triangles). Training was performed while stimulation was applied (filled symbols), while retention tests at day 1 (RT-D1-1), day 2 (RT-D2-1…RT-D2-3) and 7 (RT-D7-1…RT-D7-3) were performed without stimulation (open symbols). * indicates blocks where LSD post hoc tests indicate significant differences of anodal tDCS versus sham stimulation (p < 0.001). Data are shown as M ± SEM.

Next we investigated whether training with anodal tDCS influenced performance gains or retention differently than training with sham tDCS. Performance improvements relative to the first practice block (train1) are shown in Fig 3. Learning occurred in both sessions (main effect of *block*: F(15,180) = 12.89, p < 0.0001) and differences in learning gains during training (solid symbols) were minor when compared between anodal tDCS and sham tDCS sessions (main effect of *stimulation*: F(1, 12) = 1.598, p = 0.230). However, during the retention tests (performed without stimulation), performance in the two sessions started to differ. RT performance following training with anodal tDCS was better than RT performance following training with sham tDCS, an effect that reached significance at RT-D7 (*block* x *stimulation* interaction: F(15, 180) = 3.21, p < 0.001).

**Fig 4:**
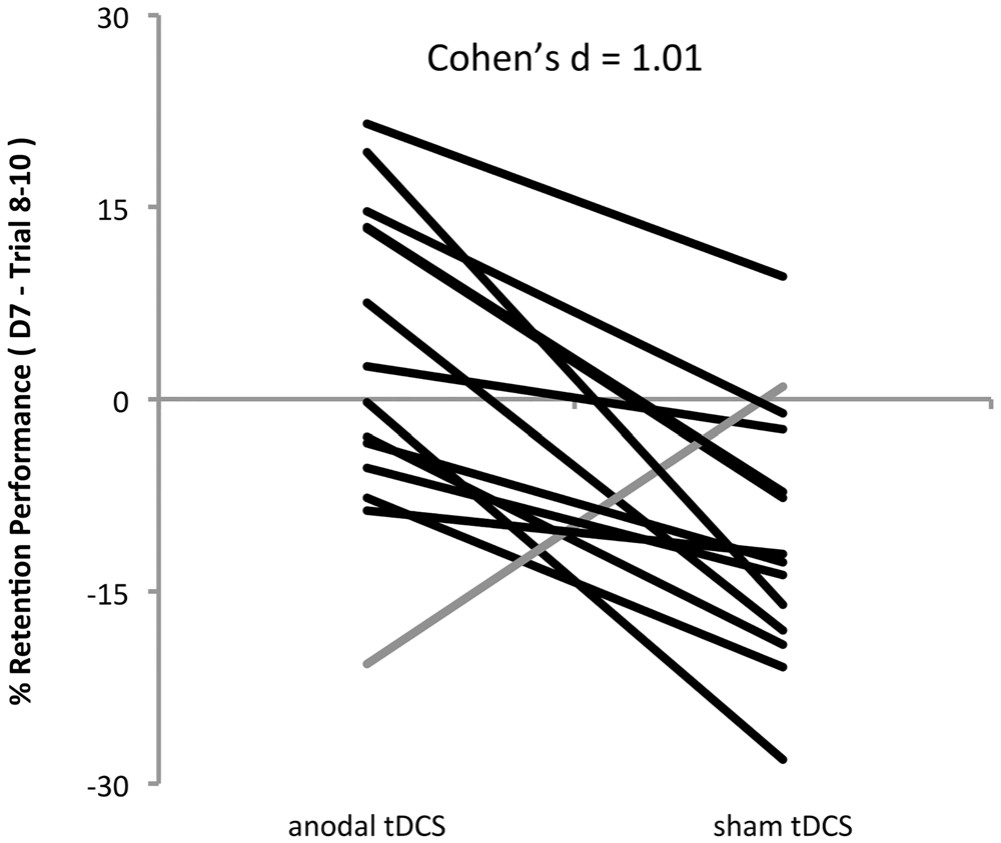
Individual subject data. Individual subject data showing gains/savings measured during the retention test at day 7 compared to performance at the end of training (i.e. average performance at RT-D7-1…3 minus average performance at train8…10). Individuals exhibiting the same trend as the group average are shown in black. Only one subject (gray) exhibited better retention performance after practice with sham tDCS than after practice with anodal tDCS.

We further investigated whether the effect of anodal tDCS during training on retention performance one week later (RT-D7) was consistent across individuals. Performance savings/gains for the anodal tDCS (**Δ**anodal) and sham tDCS (**Δ**sham) sessions were calculated at the single subject level by subtracting the average % performance improvement at the end of training (i.e. the average of practice blocks train8…train10) from the average % performance improvement at RT-D7 (i.e. the average of RT-D7-1…3). Fig 4 shows that all but one participant had larger savings/gains when they trained with anodal tDCS than when they trained with sham tDCS. Note, however, that there were large individual differences whether participants exhibited performance gains (i.e. better performance at RT-D7 than at train8…train10) or losses (i.e. worse performance at RT-D7 than at train8…train10). Effect size was calculated by dividing the mean of individual differences between gains/losses of the anodal tDCS versus sham tDCS session by the standard deviation, i.e.

$$Cohen\prime s\;d=\frac{mean(\Delta anodal_{i}-\Delta sham_{i})}{stdev(\Delta anodal_{i}-\Delta sham_{i})}$$

with *i* indexing all individual subjects, yielding a Cohen’s d of 1.01, i.e. anodal tDCS had a *large* effect on retention performance.

**Fig 5:**
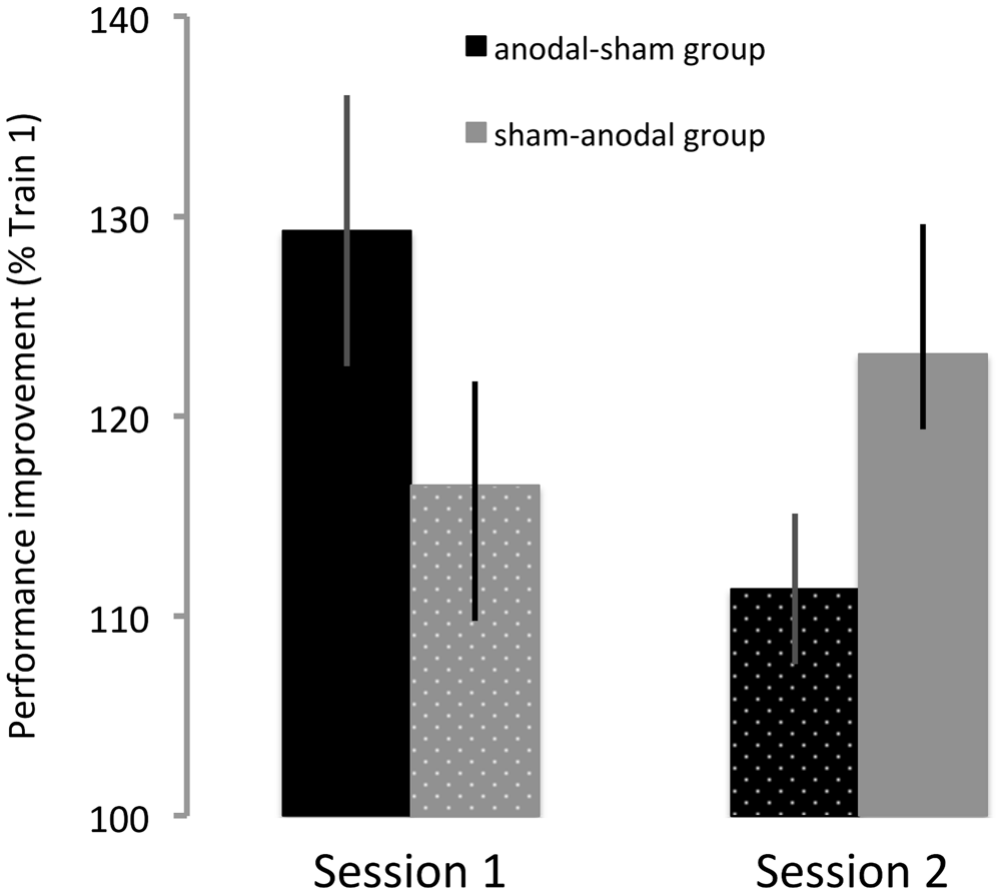
Significant *session x order* interaction. Individuals in the anodal—sham group (black, n = 7) exhibited larger performance improvements in the first than in the second session. By contrast, individuals in the sham—anodal group (gray, n = 7) exhibited smaller performance improvements in the first than in the second session. This finding lends further support to the observation that practice with anodal tDCS (solid bars) facilitated learning in comparison to sham tDCS (dotted bars). Data are shown as M ± SEM.

There was also a significant *stimulation* x *order* interaction effect (F(1,12) = 7.44, p = 0.018) that is the result of subjects in the anodal-sham group exhibiting larger overall performance improvements in the first than in the second session, while subjects in the sham-anodal group improved more in the second than in the first session (Fig 5). This confirms that differences in performance improvement between sessions resulted from the stimulation condition rather than from unspecific order effects.

## Discussion

In the present study we induced use-dependent plasticity through repetitive motor practice and used this phenomenon as a model to study the influence of anodal versus sham tDCS on LTP-like synaptic plasticity in human M1. Our main result is that retention performance was significantly better when anodal tDCS was applied during training when compared to training with sham tDCS. Significance was only reached when retention was tested one week after training, however, the size of this effect was large (Cohen’s d = 1.01) and consistent across subjects (13 out of 14 subjects followed the trend at the group level).

Our finding that anodal tDCS facilitated motor memory formation, but that its beneficial effect was mainly expressed in a retention test, is in line with previous work [9,18,40–46]. In particular Reis at al., [41] observed that when tDCS was applied to M1 during visuomotor adaptation, performance benefits were only found 3 h after the end of training. Similar to the present study they also found that no additional performance gains were observed when retention was tested after a single night of sleep, suggesting that the beneficial effect of anodal tDCS on memory consolidation is not sleep dependent. Moreover, Reis et al., [41] reported that beneficial effects were not found when stimulation was applied after practice, suggesting that the simultaneous application of anodal tDCS and practice triggers subsequent processes important for motor memory formation. Our study confirms and extends this research by demonstrating that anodal tDCS modulates the long-term effects of use-dependent plasticity, a phenomenon that is believed to be mediated by strengthening synapses via a LTP-like process [26,27,29].

One has to note, however, that polarity specific effects of tDCS on neuroplasticity might differ across brain areas. For example, Peters et al., [47] demonstrated that applying anodal tDCS over the primary visual cortex during perceptual learning blocks rather than facilitates memory consolidation. Thus, the plasticity enhancing effect of anodal tDCS reported here might be specific to the motor cortex.

In our study it is surprising that the strongest effects were found when retention was tested one week after training. Note that the D2 retention test consisted of 3 practice blocks which resulted in highly significant performance improvements regardless of whether initial training was performed with anodal or sham tDCS (separate repeated measures ANOVA with the factor *block* (D2-RT1…3: F(2,11) ≥ 13.82, p ≤ 0.001). In other words, D2 was not only a retention test but also a second training session. A clear dissociation in performance between anodal and sham tDCS training only occurred after the D2 practice blocks were finished and retention was tested again at D7. More specifically, following the anodal tDCS session the performance level reached at the end of D2 was largely maintained when long term retention was tested at D7, while it was nearly completely forgotten when initial training occurred with sham tDCS. This pattern of results suggests that combining anodal tDCS with training during D1 might have upregulated plasticity mediating mechanisms for approximately 24 hours, which in turn led to subsequent practice at D2 resulting in better long term memory formation. Indirect support for this proposal comes from Monte-Silva et al., [48] who showed that when anodal tDCS was applied at rest (i.e. during two 13 min sessions separated by 3 or 20 min), corticomotor excitability in M1 remained elevated for more than 24 hours after stimulation. Thus, in principle, tDCS effects can outlast stimulation by more than 24 hours, however, future research is required to confirm whether a similar principle is also applicable when anodal tDCS is combined with motor training because the interaction between plasticity inducing brain stimulation and training is complex, non-additive [49] and might be influenced by homeostatic principles [50].

In the present study we used a within subject cross-over design which is advantageous because it controls for inter-individual variability in ability to improve performance due to practice, which can be large [36] and might mask the modulatory effects of tDCS. Additionally, this design can be used to quantify the effect of anodal tDCS on memory formation at the level of the individual. Interestingly, in our study all but one subject had better retention performance in the anodal tDCS session than in the sham tDCS session, and the effect was large (Cohen’s d = 1.01) indicating that our sample responded very consistently to the experimental manipulation.

Our study also has several limitations. First, based on previous work one would expect that the effect of tDCS on motor memory formation might be more reliably measured when it accumulates over multiple sessions [40,41,51,52]. We used a single practice session that was followed by several retention tests because we were concerned that too much training would lead to highly automatized performance during the first session, which would lead to either no or significantly less learning gains during the second session. With our rather short practice period we were indeed able to show that performance was generally better in the second than in the first session, but that learning gains were comparable and did not differ significantly. Second, we used a relatively simple motor task that might, in theory, cause ceiling effects in the response to anodal tDCS [53,54]. It is possible that the tDCS effect size is even bigger for more complex skills. Finally, we did not stimulate a control region, and thus cannot provide any insight into the anatomical specificity of the effect.

In summary our data strongly suggest that anodal tDCS facilitates long term memory formation reflecting use-dependent plasticity in the motor cortex. We used this task because previous research has convincingly demonstrated that performance changes reflect synaptic plasticity mediated by an LTP-like mechanism and, in line with this work, our results suggest that anodal tDCS might facilitate these processes.

## Supporting Information

S1 File Normalized performance data (tab separated txt file). (TXT)
